# ARHGAP42 promotes cell migration and invasion involving PI3K/Akt signaling pathway in nasopharyngeal carcinoma

**DOI:** 10.1002/cam4.1552

**Published:** 2018-06-24

**Authors:** Qian Hu, Xiao Lin, Linxiaoxiao Ding, Yinduo Zeng, Danmei Pang, Nengtai Ouyang, Yanqun Xiang, Herui Yao

**Affiliations:** ^1^ Guangdong Provincial Key Laboratory of Malignant Tumor Epigenetics and Gene Regulation Sun Yat‐Sen Memorial Hospital of Sun Yat‐Sen University Guangzhou China; ^2^ Department of Oncology Sun Yat‐Sen University Cancer Center Guangzhou China; ^3^ Department of Breast Cancer Oncology Foshan Hospital of Sun Yat‐Sen University Foshan China; ^4^ Department of Breast Cancer Oncology Sun Yat‐Sen Memorial Hospital of Sun Yat‐Sen University Guangzhou China; ^5^ Department of Nasopharyngeal Carcinoma Sun Yat‐Sen University Cancer Center Guangzhou China

**Keywords:** ARHGAP42, metastasis, nasopharyngeal carcinoma, uc010rul

## Abstract

Rho GTPase‐activating protein 42 was identified as an inhibitor of RhoA to maintain normal blood pressure homeostasis. However, the effect of ARHGAP42 in promoting cell malignancy in nasopharyngeal carcinoma is demonstrated in this study. Microarray and real‐time quantitative PCR were used for a mRNA profiling of ARHGAP42 in nasopharyngeal primary and metastatic carcinoma tissues. Western blot and immunohistochemical staining were used for detecting the expression of ARHGAP42 protein in nasopharyngeal carcinoma tissues and cell lines. The overexpression and silence experiments of ARHGAP42 were performed in NPC cell lines using siRNA and expressive plasmid for evaluating cancer cell migration and invasion in vitro. Real‐time quantitative PCR, western blot, and transwell test were employed for with the function of ARHGAP42 and its antisense lncRNA uc010rul. We confirmed the elevated expression of ARHGAP42 in metastatic NPC tissues of mRNA and protein for the first time. Immunohistochemical analysis indicated that NPC patients with highly ARHGAP42 expression were significantly associated with shorter metastasis‐free survival. Knockdown of ARHGAP42 resulted in significant inhibition of nasopharyngeal cancer cell migration and invasion in vitro, and the overexpression of ARHGAP42 showed the opposite effects. In addition, the silence of uc010rul resulted in ARHGAP42 expression decrease and significant inhibition of nasopharyngeal cancer cell migration and invasion. High expression of ARHGAP42 is associated with poor metastasis‐free survival of nasopharyngeal carcinoma patients. ARHGAP42 promotes migration and invasion of nasopharyngeal carcinoma cells in vitro*;* the antisense lncRNA may be involved in this effect.

## 1 INTRODUCTION

Nasopharyngeal cancer (NPC) is a neoplasm of the head and neck that is prevalent in South‐East Asia.^1^, ^2^, ^3^ Although the radiotherapy can control early stage of NPC, tumor recurrence and distant metastasis are the key difficulties of NPC therapy.^4^, ^5^, ^6^, ^7^ At present, NPC tumorigenesis and progression are thought to be multistep processes involving multiple genetic and epigenetic changes.^8^, ^9^, ^10^, ^11^ Although molecular biology studies have improved general understanding of the tumorigenesis and progression of NPC, the appropriate biomarkers for metastasis mechanism have not yet been identified.

ARHGAP42 (also known as GRAF3) is a GTPase‐activating protein.^12^ Rho family small GTPases regulate cellular functions, including diverse cytoskeleton‐related events, gene transcription, and immune cell migration and inflammation.^13^ GTPase activator proteins (Rho/Rac GAPs) increase the GTP hydrolysis, which drove them to an inactive state.^14^, ^15^ This step is a key process of signal termination.^16^ The Rho GTPase‐activating proteins (Rho‐GAPs) are one of the most important types of regulators of Rho GTPases.^16^ A previous study has identified ARHGAP42 as a Rho‐specific GAP expressed specifically in smooth muscle cells (SMCs) in mice and humans.^12^ The study has indicated that ARHGAP42‐mediated inhibition of RhoA activity in vascular SMCs is necessary to maintain normal blood pressure homeostasis. Rho GTPases are crucial in cytoskeletal organization, cell growth, differentiation, and neuronal functions. However, little is known about the role of ARHGAP42 in cancer development.

## 2 MATERIALS AND METHODS

### 2.1 Patient and tissue specimens

A total of 85 NPC RNA sample and 104 paraffin‐embedded NPC samples were examined in this study. The patients accepted into this study were diagnosed with NPC at the Sun Yat‐Sen cancer center. Written informed consents were obtained from all patients, and the protocol was approved by the Research Ethics Board at Sun Yat‐Sen Memorial Hospital. The clinical information is described in detail in Supporting Information. All of patients had received no therapy prior to biopsy. The tumors were classified in accordance with the 92‐classification system.

### 2.2 LncRNA and mRNA expression profiling

To focus on those clinically relevant mRNA, we used Arraystar Human lncRNA/mRNA Expression Profiling Service Report in KANG CHEN company. For microarray analysis, Agilent Array platform was employed for four primary tumor tissues and three metastatic tissues from the patients with nasopharyngeal carcinoma. The sample preparation and microarray hybridization were performed based on the manufacturer's standard protocols with minor modifications. To brief, mRNA was purified from total RNA after removal of rRNA (mRNA‐ONLY^™^ Eukaryotic mRNA Isolation Kit, Epicentre). Quantile normalization and subsequent data processing were performed using the GeneSpring GX v11.5.1 software package (Agilent Technologies). After normalization of the raw data, mRNAs that have flags in Present or Marginal at least seven of seven samples were chosen for further data analysis. Differentially expressed mRNAs with statistical significance between the two groups were identified through Volcano Plot filtering. At last, hierarchical clustering was performed to show the distinguishable mRNA expression pattern among samples.

### 2.3 Immunohistochemistry

The specimens were paraffin‐embedded. After dewaxing and rehydration, the slides were washed with PBS. After heating, 1% hydrogen peroxidase was used to block endogenous peroxidase activity. The specimens were then incubated with anti‐ARHGAP42 (1:50, Abcam, USA) for 90 minutes at room temperature. After washing with PBS, the samples were incubated with secondary antibodies for 60 minutes at room temperature. At last, DAB solution was used for colorization and followed by counterstaining of all sections with hematoxylin. We evaluate the staining by calculating the average positively stained tumor cells under microscope by two researchers independently and blindly to the clinical parameters.

### 2.4 Cell lines

NPC cell lines CNE1, CNE2, S18, S26, C666‐1, and immortalized nasopharyngeal epithelial cell NP69 were obtained from ATCC. These cancer cells were maintained in RPMI‐1640 supplemented with 10% fetal calf serum, 5% CO_2_ at 37°C. And the immortalized nasopharyngeal epithelial cell NP69 was maintained in K‐SFM with EGF and BPE (Invitrogen Life Technologies, Carlsbad, CA, USA.)

### 2.5 siRNA transfection

The cells were seeded into six‐well plates at 2 × 10^5^ cells per well, grown for 24 hours in complete medium and then transfected with Lipofectamine 2000 (Invitrogen Inc.) and siRNA specific to ARHGAP42 or siRNA‐negative control that designed and synthesized by Gene Pharma (Shanghai, China). RNA primers complementary to nucleotides were used for silence. The sequences of siRNA nucleotides are shown in Supporting Information. At a final concentration of 5 Nm was used according to the manufacturer's directions. To check the effect of knockdown for LncRNA and mRNA, cells were harvested 2 days after siRNA transfection and analyzed by RT‐qPCR and western blot as described below. Three days after transfection, cells were detached and used for proliferation, migration, and invasion assays, as described below.

### 2.6 Real‐time quantitative PCR

Total RNA from specimens or cell lines was extracted with the Trizol reagent (Invitrogen Life Technologies, Carlsbad, CA, USA) according to the manufacturer's instructions, and cDNA was obtained by reverse transcribing the total 500 ng RNA with a Reverse Transcription Kit (Takara Inc, USA). GAPDH was used as an internal control, and gene values were normalized to GAPDH. Real‐time PCR was performed on a Roche Light Cycler480 Real‐Time PCR System. Primer sets are listed in Supporting Information. Three biological replicates were used for quantification analysis, and three technical replicates were analyzed for each biological replicate.

### 2.7 Western blot

Cell lysates were prepared by a RIPA lyses solution. Protein concentration was measured using the BCA protein assay kit (PIERCE, Rockford, IL).After equal amount of protein was resolved on 12% SDS‐PAGE, the proteins were electrotransferred from gel to PVDF membrane. The membrane was blocked with 5% nonfat milk solution for 1 hour and incubated with primary monoclonal antibody against the target protein overnight at 4°C overnight. Anti‐GAPDH (1:1000, Santa Cruz, CA, USA) was used as an internal control. The membrane was subjected to immunoblotting using HRP‐conjugated antibody. The membrane was detected by the enhanced chemiluminescence (ECL) detection system.

### 2.8 Cell proliferation assay

The cells were then seeded in six‐well plates (5 × 10^4^ cells/mL) with 2 mL cell suspension in each well. After transfection, the cells were seeded in the 12‐well plates. After 72 hours, we counted the cell number through cell counter. Each experiment was repeated three times.

### 2.9 Migration and invasion assay

Transwell assays were performed in modified Boyden chambers with 8‐mm pore filter inserts in 24‐well plates (Corning). In brief, 1 × 10^5^ cells in RPMI 1640 medium with 0.2% bovine serum albumin were added to the inserts of a 24‐well culture plate. RPMI 1640 medium with 10% FCS was added to the lower chambers. After 8 hours, the nonfiltered cells on the upper side of the insert were gently removed with cotton swabs. The cells that had passed through the filters to the lower sides of the insert were stained with crystal violet and photographed.

### 2.10 Statistical analysis

All analyses were carried out using the SPSS 11.0 software. The chi‐square tests for proportion were used to analyze the relationship between ARHGAP42 expression and clinicopathologic characteristics. Survival curves were plotted by the Kaplan‐Meier method and compared by the log‐rank test. A *P* value <.05 was considered statistically significant.

## 3 RESULTS

### 3.1 ARHGAP42 mRNA is highly expressed in profiling of NPC

**Figure 1:**
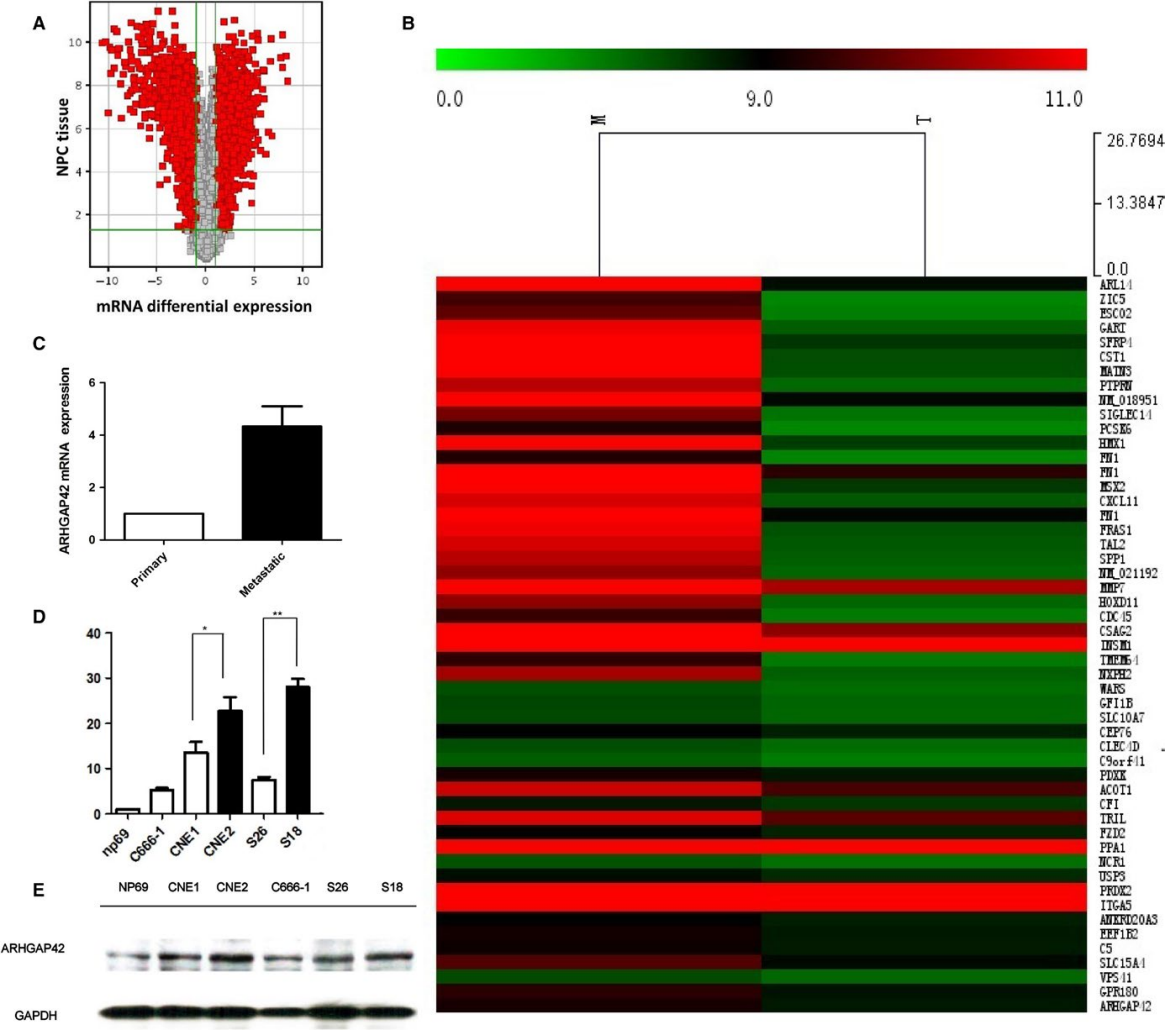
ARHGAP42 is highly expressed in NPC cell lines. A, Volcano plots based on mRNA profiling in four primary tissues compared with three metastatic tissues from patients with nasopharyngeal carcinoma showed mRNA differential expression. B, Hierarchical clustering was performed based on mRNA profiling in four primary NPC tissues compared with three metastatic tissues from patients with nasopharyngeal carcinoma showed mRNA differential expression. C, Microarray validation for upregulation of ARHGAP42 in NPC and metastatic tissues by qPCR. D, ARHGAP42 mRNA highly expressed in NPC cell lines, C666‐1, CNE1, CNE2, S26, S18, 5‐8F, and 6‐10B cells measured by quantitative real‐time PCR, normalized to GAPDH gene expression, compared by NP69, especially in highly metastasis potential cell lines, S18 and 5‐8F. E, ARHGAP42 protein highly expressed in NPC cell lines, C666‐1, CNE1, CNE2, S26, and S18 analyzed by western blotting with antibodies against ARHGAP42, especially in highly metastasis potential cell line S18

First, we performed RNA profiling to identify the altered RNAs in the NPC tissue specimens. We used Arraystar RNA Expression Profiling Service Report in KANG CHEN Company by four primary NPC tissues and three metastatic tissues. The mRNA profiling results are shown in Figure 1A as Volcano Plot. Of 30 215 cDNA probes, 1787 genes were differentially expressed (twofold), 199 genes being upregulated more than 10‐fold, and only 71 genes being upregulated more than 30‐fold in metastatic NPC tissues. Among them, ARHGAP42 is most abnormally expressed and selected for validation by qRT‐PCR. Microarray validation for upregulation of ARHGAP42 in NPC and metastatic tissues by qPCR is showed in Figure 1C. In metastatic tissues, ARHGAP42 expression is twofold than primary NPC tissues. The results in Figure 1D show that ARHGAP42 was confirmed to be upregulated in NPC cell lines. The high expression of ARHGAP42 protein is also confirmed in NPC cell lines including C666‐1, CNE1, CNE2, S26, and S18 by western blotting as shown in Figure 1E.

### 3.2 ARHGAP42 is associated with poorer survival of patients with NPC

**Figure 2:**
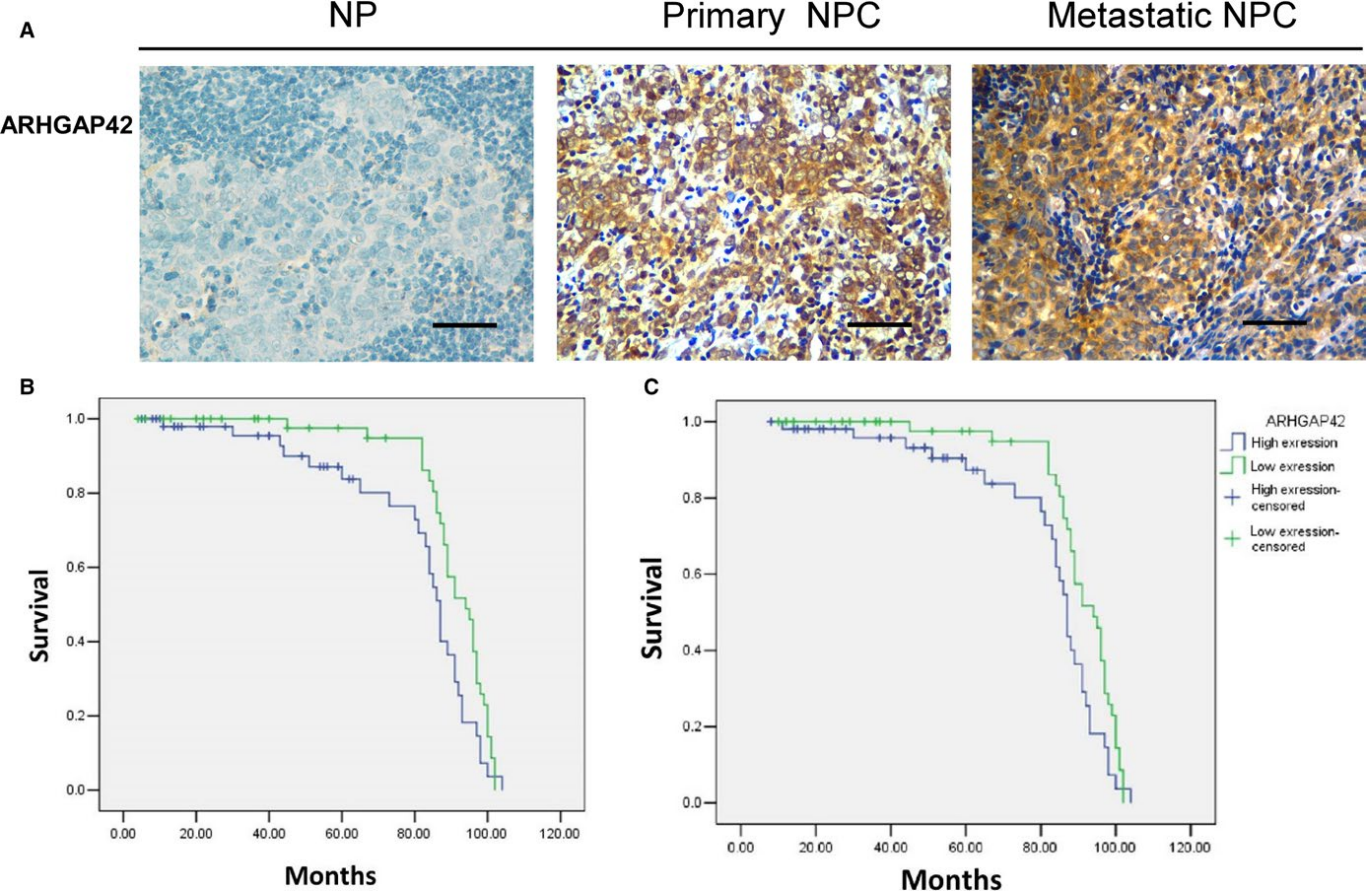
ARHGAP42 is highly expressed in NPC tissues. A, ARHGAP42 IHC staining in NPC tissue. Representative immunohistochemical expression of ARHGAP42 immunostaining in NP and NPC primary and metastatic tissues. Brown pointing at a dermal nest of stained cells (×400), scale bar: 50 μm. B, Survival analyses were performed in a cohort of 104 patients. Shorter OS was observed in the patients with primary NPC expressing high levels of ARHGAP42 proteins. C, Survival analyses were performed in a cohort of 104 patients. Shorter MFS was observed in the patients with primary NPC expressing high levels of ARHGAP42 proteins

We also observed ARHGAP42 overexpression in NPC tissues. In comparison, ARHGAP42 protein is significantly higher in NPC tissues, than normal nasopharyngeal epithelium as shown in Figure 2A. Furthermore, ARHGAP42 protein is significantly higher in metastatic NPC tissues than primary NPC tissues as seen in Figure 2A. We next analyzed the correlation between the expression of ARHGAP42 protein and clinicopathological characteristics of NPC. As summarized in Table 1, there are no significant correlations between ARHGAP42 expression and gender, age, pathology classification, N classification, T classification, and TNM classification. However, ARHGAP42 overexpression is positively associated with distant metastasis as described in Table 2. In an interesting manner, we found that the overexpression of ARHGAP42 in primary tumors is significantly correlated with overall survival of patients with NPC. To evaluate the association between ARHGAP42 expression and patient's outcome, Kaplan‐Meier curves were plotted. As shown in Figure 2B,C, Tables 3 and 4, patients with high ARHGAP42 expression have significantly shorter overall and metastasis‐free survival rates compared to those with low ARHGAP42 expression.

**Table 1:** Four NPC primary tissues and three metastatic tissues from the patients with nasopharyngeal carcinoma subjected to microarray analysis

Patient	Gender	Pathology	TNM staging	EBV‐DNA (/copies)
A	Male	Primary undifferentiated carcinoma	T3N2M0	0
B	Male	Primary undifferentiated carcinoma	T3N2M0	4.7 × 10^6^
C	Female	Primary undifferentiated carcinoma	T3N2M0	0
D	Male	Primary undifferentiated carcinoma	T3N3M0	1.0 × 10^6^
E	Male	Dorsum metastatic Undifferentiated carcinoma	IV	1.1 × 10^6^
F	Male	Shoulder metastatic Undifferentiated carcinoma	IV	NA
H	Male	Sternail metastatic squamous carcinoma	IV	0

**Table 2:** Relationship between the expression of ARHGAP42 and clinicopathological characteristics in NPC

Parameter	Group	N	ARHGAP42 expression		Chi‐square value	*P* value
			Low	High		
Gender	Male	81	40	41	0.365	.639
	Female	23	13	10		
Age	>50	39	18	21	0.577	.544
	≤50	65	35	30		
WHO PS	0‐1	76	40	36	0.315	.660
	>2	28	13	15		
TNM staging	Stage I‐II	46	26	20	1.020	.331
	Stage III‐IV	58	27	31		
Tumor staging	I‐II	59	32	27	0.585	.553
	III‐IV	45	21	24		
Lymph node invasion	NO	80	41	39	0.012	1.00
	YES	24	12	12		
Metastasis	NO	88	49	39	5.1	.03*
	YES	16	4	12		
Relapse	NO	84	45	39	3.591	.081
	YES	20	6	14		
Pathology	U	94	47	47	0.362	.742
	D	10	6	4		
Concurrent chemotherapy	NO	94	45	49	0.012	1.00
	YES	10	6	4		
Induced chemotherapy	NO	81	41	39	0.012	1.00
	YES	23	12	12		

D, differentiated nonkeratinized carcinoma; U, undifferentiated nonkeratinized carcinoma (* P<0.05).

**Table 3:** Expression of ARHGAP42 is correlated with the overall survival of 104 patients with NPC

Group	OS (mo)			
	Mean	Standard error	95% CI	
			Inferior	Upper
Low expression	91.182	1.761	87.730	94.634
High expression	82.318	2.982	76.473	88.163
Total	87.048	1.770	83.380	90.517

**Table 4:** Expression of ARHGAP42 is correlated with the metastasis‐free survival of 104 patients with NPC

Group	MFS (mo)			
	Mean	Standard error	95% CI	
			Inferior	Upper
Low expression	91.066	1.831	87.426	94.655
High expression	80.681	3.307	74.200	87.162
Total	86.311	1.910	82.568	90.054

### 3.3 ARHGAP42 siRNA inhibits cell proliferation and mobility

**Figure 3:**
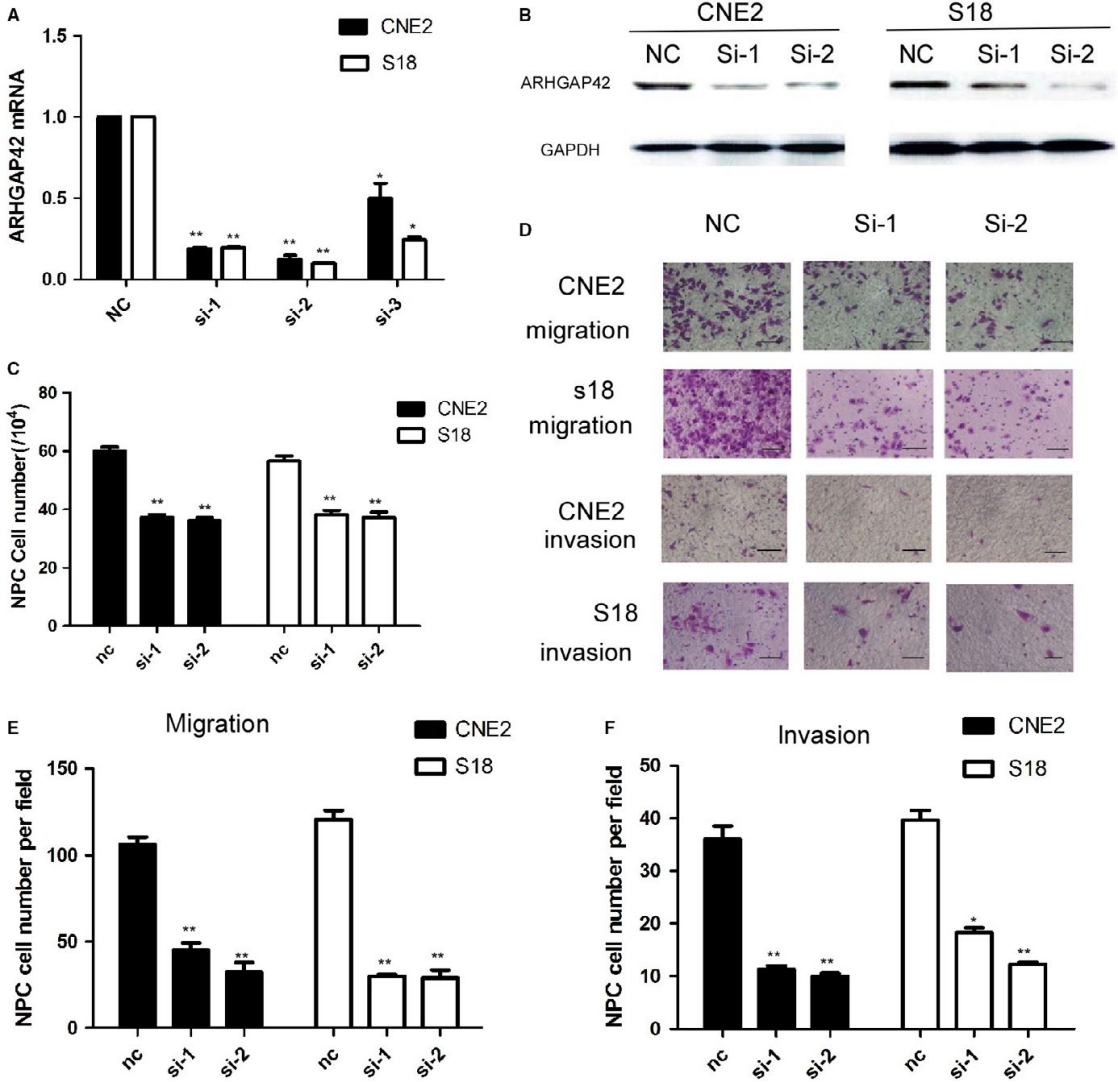
Knocking down of ARHGAP42 by siRNA resulted in significant inhibition of cell proliferation and mobility inhibition of S18 and CNE2. A, ARHGAP42 mRNA expression in the NPC cell lines CNE2 and S18 by siRNA‐ARHGAP42 was measured by quantitative real‐time PCR, normalized to GAPDH gene expression. *P < .05, **P < .01. B, ARHGAP42 protein expression in the NPC cell lines CNE2 and S18 by siRNA‐ARHGAP42 was measured by western blotting. C, Knocking down of ARHGAP42 by siRNA resulted in significant inhibition of S18 and CNE2 cell proliferation by cell counting test in 72 h. *P < .05, **P < .01, compared with the negative control group. D, Knocking down of ARHGAP42 by siRNA resulted in significant moveability inhibition of S18 and CNE2 cell lines, scale bar:50 μm. E, Knocking down of ARHGAP42 by siRNA resulted in significant inhibition of S18 and CNE2 cell migration. **P < .01, compared with the negative control group. F, Knockdown of ARHGAP42 by siRNA resulted in significant inhibition of S18 and CNE2 cell invasion

To examine the functions of ARHGAP42 in NPC cells, S18 and CNE2 cells are collected following transfection with ARHGAP42 siRNA or control siRNA. The levels of mRNA and protein of ARHGAP42 are significantly decreased in CNE2 and S18 cells that are transfected with siRNA as shown in Figure 3A,B (*P* < .01). ARHGAP42 siRNA inhibits CNE2 and S18 cell growth after 72‐hour incubation as seen in Figure 3C (*P* < .01). The cell migration and invasion are decreased after transfection with ARHGAP42 siRNA in both CNE2 and S18 cell lines, respectively, as shown in Figure 3E,F (*P* < .01).

### 3.4 Overexpression of ARHGAP42 promotes cell proliferation and mobility

**Figure 4:**
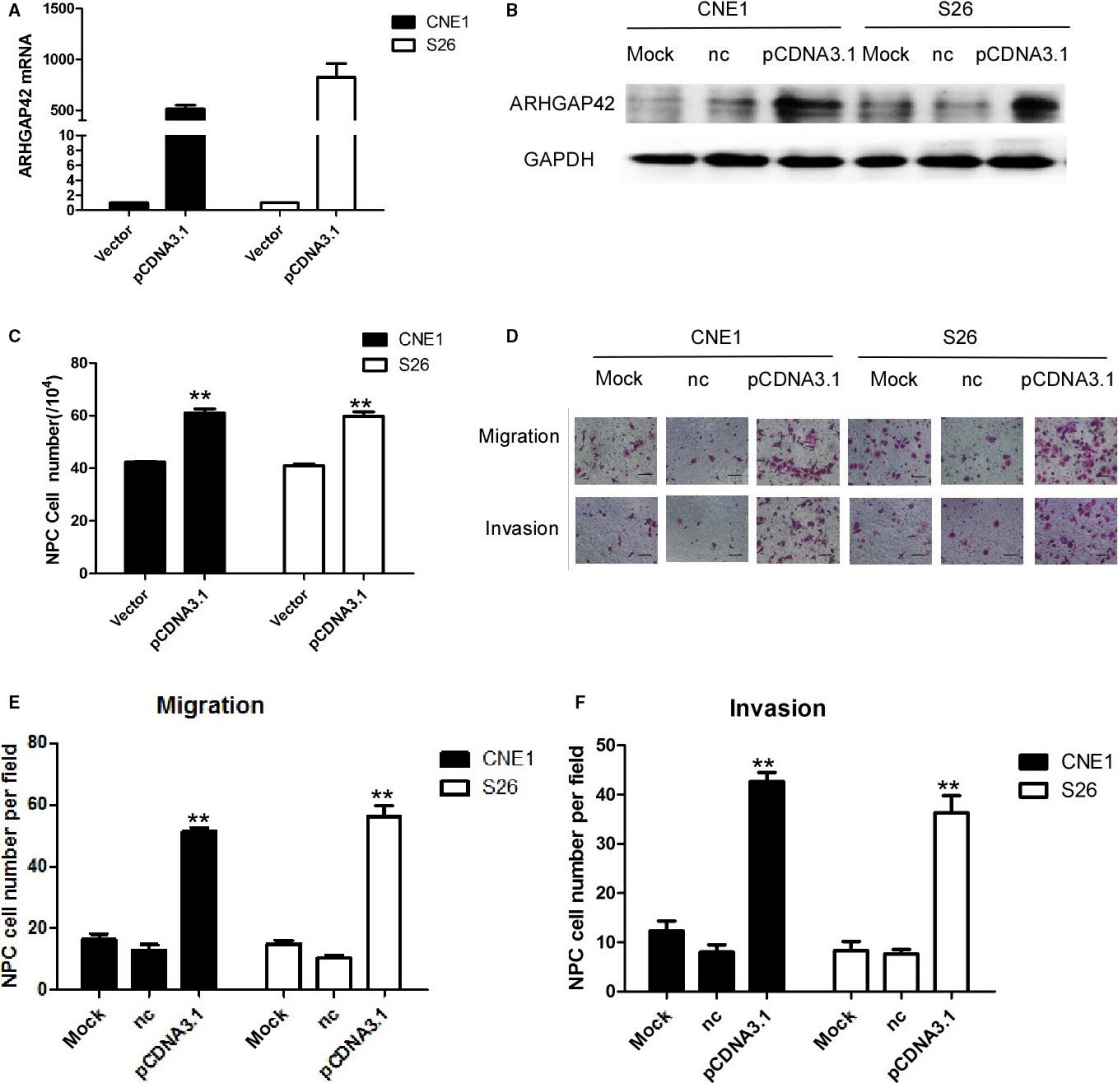
ARHGAP42 promotes cell proliferation and mobility. A, Overexpression of ARHGAP42 mRNA was achieved in S26 cells and CNE1 cells by transfection of the ARHGAP42 expression vector. B, Overexpression of ARHGAP42 protein was achieved in S26 cells and CNE1 cells by transfection of the ARHGAP42 expression vector. C, The cell counting test showed CNE1 and S26 cells were significantly increased after ARHGAP42 overexpression. D, The migration and invasion of S26 cells were significantly increased after ARHGAP42 overexpression, scale bar:50 μm. E, Overexpression ARHGAP42 resulted in significant inhibition of S18 and CNE2 cell migration. F, Overexpression ARHGAP42 resulted in significant inhibition of S18 and CNE2 cell invasion

To further examine the functions of ARHGAP42 in NPC cells, S26 and CNE1 cells are collected following transfection with ARHGAP42‐pcDNA3.1 vector or control vector. The low‐invasive NPC cell lines CNE1 and S26 that transient transfected with ARHGAP42‐pcDNA3.1 vector show higher ARHGAP42 expression than that with control vector. As demonstrated by real‐time qPCR analysis, the transcription and protein level of ARHGAP42 are significantly increased in CNE1 and S26 cells that transfected with ARHGAP42 vector as indicated in Figure 4A (*P* < .01). ARHGAP42 protein level is significantly increased in both CNE1 and S26 cell lines that transfected with ARHGAP42‐pcDNA3.1 vector as shown in Figure 4B. ARHGAP42 overexpression promotes the growth of CNE1 and S26 cell growth, migration, and invasion in CNE1 and S26 cell lines after incubation 72 hours as shown in Figure 4C (*P* < .01), as well as enhances NPC moveability as seen in Figure 3D‐F (*P* < .01).

### 3.5 uc010rul is in association with NPC cell migration and invasion

**Figure 5:**
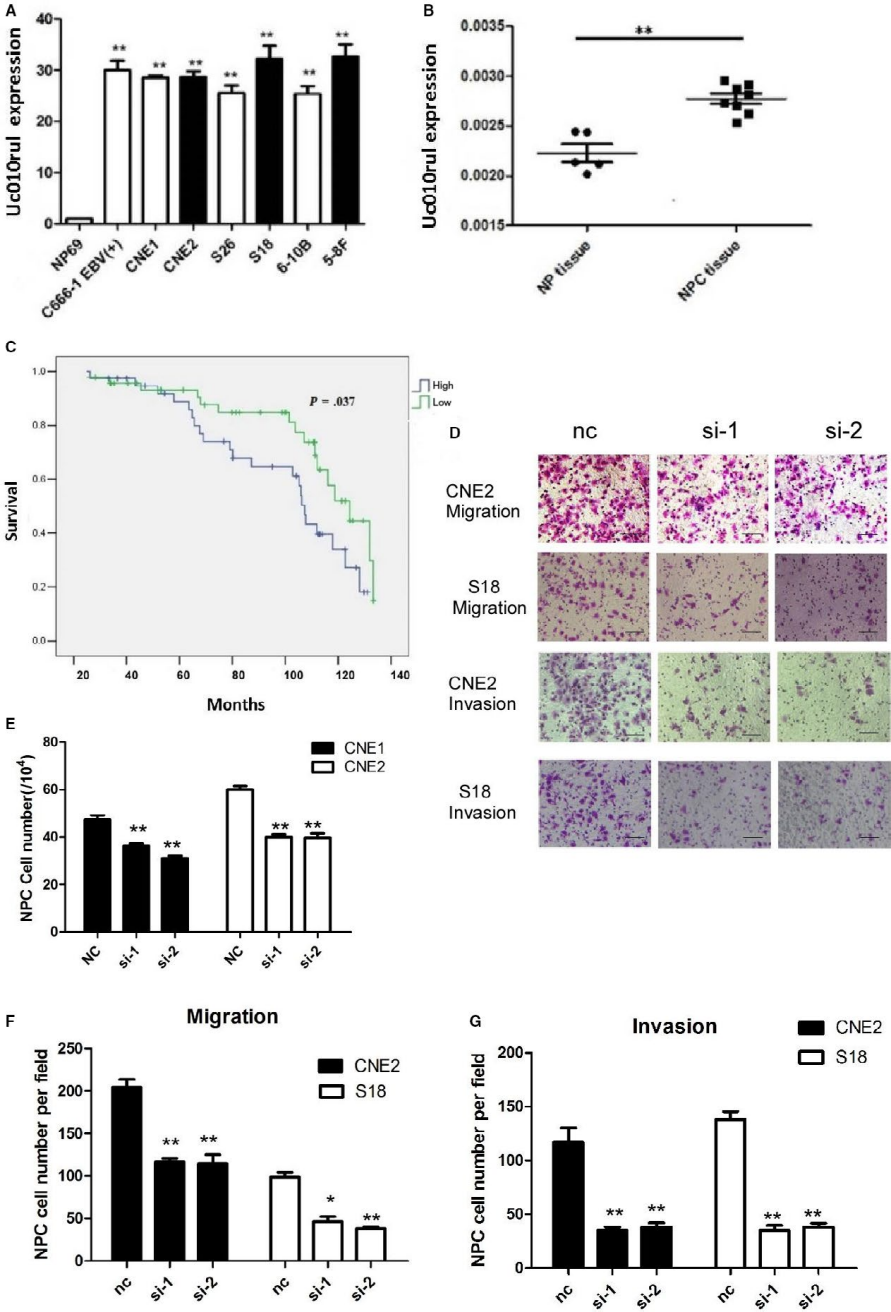
Antisense lncRNA uc010rul is associated with the mobility of NPC cells. A, LncRNA uc010rul expression in the NPC cell lines was measured by quantitative real‐time PCR, normalized to GAPDH gene expression. B, LncRNA uc010rul expression in NPC or normal adjacent tissues was measured by quantitative real‐time PCR, normalized to GAPDH gene expression. *P < .05, **P < .01. C, Survival analyses were performed in a cohort of 85 patients. A shorter OS was observed in patients with primary NPC expressing high levels of uc010rul. *P < .05. D, Knocking down of uc010rul by siRNA resulted in S18 and CNE2 cell moveability decreased, scale bar: 50 μm. E, LncRNA uc010rul expression in NPC cell lines CNE1 and CNE2 by siRNA‐uc010rul was measured by quantitative real‐time PCR and normalized to GAPDH gene expression. *P < .05, **P < .01. F, Silence of uc010rul resulted in S18 and CNE2 cell migration ability inhibited. *P < .05, **P < .01, compared with the negative control group. G, Silence of uc010rul resulted in S18 and CNE2 cell invasion ability inhibited. **P < .01, compared with the negative control group

To explore the role of antisense lncRNA uc010rul in NPC, uc010rul was overexpressed in S18 and CNE2 as shown in Figure 5A. Uc010rul expression is detected in 10 NPC tissues along with five normal tissues and several NPC cell lines. uc010rul expression in the NPC tissues is significantly higher than that in the control group as indicated in Figure 5B (*P* < .01). The patients who have uc010rul overexpression show a significantly shorter overall and metastasis‐free survival compared to those with low uc010rul expression as seen in Figure 5C. The interfering RNA has been widely used to knock down the expression of target genes because of its high specific and low toxicity. To examine the functions of uc010rul, S18 and CNE2 cells were collected following transfection with uc010rul siRNA or control siRNA. As demonstrated by real‐time qPCR analysis, uc010rul transcription level was significantly decreased in the cells that transfected with siRNA. The RNA level of uc010rul was significantly decreased after transfection with siRNA. Furthermore, uc010rul siRNA inhibits the growth of CNE2 and S18 cells as shown in Figure 5E. The depletion of uc010rul inhibits NPC cell migration and invasion as shown in Figure 5D,F,G.

### 3.6 Overexpression of ARHGAP42 rescues the mobility and proliferation caused by uc010rul siRNA

**Figure 6:**
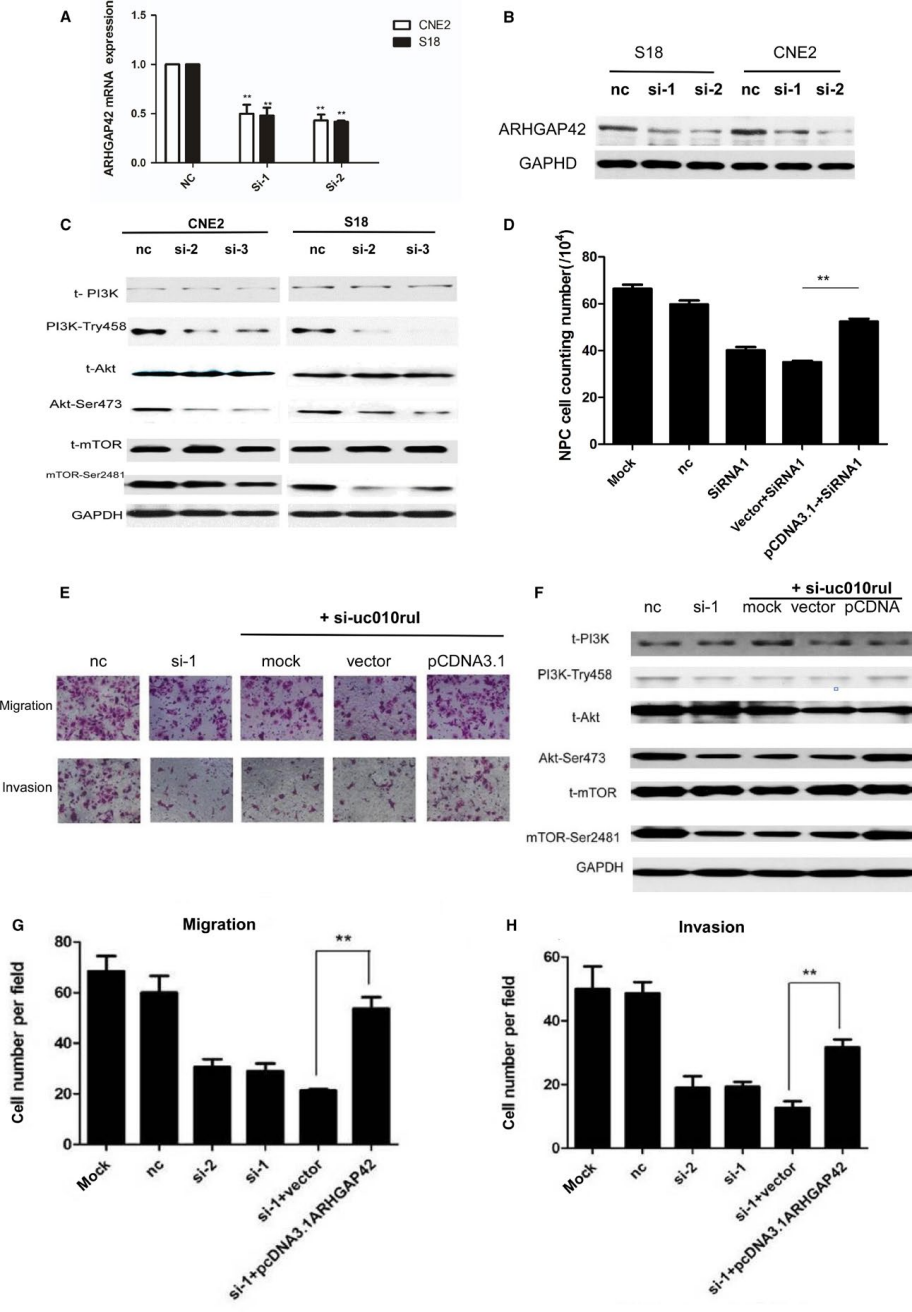
Overexpression of ARHGAP42 rescues the mobility and proliferation caused by uc010rul siRNA. A, RT‐PCR and western blotting showed that the inhibition of ARHGAP42 by specific siRNA led to no change in the expression of uc010rullncRNA in S18 and CNE2 cells. B, Western blotting showed that uc010rul‐siRNAs effectively reduced ARHGAP42 protein expression in CNE2 and S18. C, Western blotting showed uc010rul‐siRNAs effectively reduced p‐PI3K, p‐Akt, p‐mTOR protein expression in CNE2. D, The cell counting assay showed that the overexpression of ARHGAP42 rescued the proliferation inhibition caused by uc010rul siRNA in CNE2 cells. E, The migration and invasion assay showed that the overexpression of ARHGAP42 rescued the mobility decrease caused by uc010rul knockdown in CNE2 cells, scale bar: 50 μm. F, The western blotting results showed that the overexpression of ARHGAP42 rescued the PI3K, Akt, and mTOR protein phosphorylation inhibition caused by uc010rul siRNA in CNE2 cells. G, Overexpression of ARHGAP42 rescued the migration ability decrease caused by uc010rul knockdown in CNE2 cells. H, Overexpression of ARHGAP42 rescued the invasion ability decrease caused by uc010rul knockdown in CNE2 cells

We first verified the regulation role of uc010rul on ARHGAP42 by RT‐PCR and western blot. The results show that the inhibition of uc010rul by specific siRNA leads to the decrease in mRNA expression of ARHGAP42 in S18 and CNE2 cell lines in Figure 6 (*P* < .01). Therefore, ARHGAP42 may serve as a targeted gene for uc010rul. The protein level of ARHGAP42 expression in S18 and CNE2 cell lines is also decreased after inhibition of uc010rul by specific siRNA as shown in western blot results (Figure 4B). In an interesting manner, we found that p‐PI3K, p‐Akt, and p‐mTOR are significantly decreased in the cells that transfected with ARHGAP42‐siRNA (Figure 6C), implying that PI3K/Akt/mTOR signaling may be involved in the mechanism of ARHGAP42 regulating cell mobility. In order to study the role of uc010rul‐regulating ARHGAP42 in NPC cell lines, we designed the overexpression plasmid of ARHGAP42. ARHGAP42‐pcDNA3.1 vector was transient transfected into the low‐invasive NPC cell lines CNE1 and S26 which showed low ARHGAP42 expression in Figure 6. We employed the migration and invasion test, western blot, and cell counting assay. Cell counting assays also showed that ARHGAP42‐pcDNA3.1 vector was transient transfected could reverse the NPC cell lines CNE2 proliferation inhibition caused by uc010rul‐specific silence in Figure 6D. In migration and invasion test, we found that uc010rul‐siRNA‐transfected cells migrated and invaded significantly less (*P* < .01), but overexpressed ARHGAP42‐transfected reversed the effect incompletely (*P* < .01) in Figure 6E,G,H. Similar to that, uc010rul‐siRNA‐transfected cells significantly inhibited the p‐PI3K, p‐Akt, and p‐mTOR (*P* < .01), but overexpressed ARHGAP42‐transfected reversed the effect incompletely (*P* < .01) in Figure 6F. Thus, we could imply ARHGAP42 promotes migration and invasion of nasopharyngeal carcinoma cells in vitro*;* the antisense lncRNA may be involved in this effect.

## 4 DISCUSSION

Scientists have previously performed high‐throughput gene expression profiling followed by functional studies to identify and validate the important molecules that are responsible for promoting NPC metastasis, including serglycin, interleukin‐8, and HSP27y.^17^ It is also necessary to identify the specific molecular mechanisms that contribute to NPC cell metastasis. The present study analyzes the differential expression of NPC cell lines, and metastatic and primary NPC tissues. The mRNA and protein expression of ARHGAP42 are detected by RT‐PCR and western blot. We evaluated the clinicopathological significance of ARHGAP42 expression in patients with NPC. Then, CNE2 and S18 cells were transfected with ARHGAP42 siRNA, and cell proliferation and mobility were tested with cell counting test and transwell assays, respectively. CNE1 and S26 cells were transfected with ARHGAP42 overexpression vector, and cell proliferation and mobility also were tested. Furthermore, we identified the function of the antisense lncRNA uc010rul in NPC cell lines and tissues, and we explored the possible mechanisms regulation of ARHGAP42 and uc010rul. In the present study, we confirmed that ARHGAP42 was the important marker of nasopharyngeal carcinoma progression and metastasis for the first time.

This study identified a novel marker ARHGAP42, which is associated with the acquisition of NPC cell metastasis for the first time. Uc010rul, an antisense lncRNA, was also identified and confirmed to be expressed exclusively in NPC cells and was functionally characterized as a new mediator of NPC progression.

We also evaluated the clinicopathological significance of ARHGAP42 expression in patients with NPC. Rho family small GTPases regulate cellular functions, including diverse cytoskeleton‐related events, gene transcription, and immune cell migration and inflammation. GTPase activator proteins (Rho/Rac GAPs) increase the GTP hydrolysis, which drove them to an inactive state. This step is a key process of signal termination.^15^ The Rho GTPase‐activating proteins (Rho‐GAPs) are one of the major types of regulators of Rho GTPases that are crucial in cytoskeletal organization, cell growth, differentiation, and neuronal functions. ARHGAP42 (also known as GRAF3) is not a commonly studied protein. Earlier, a study has presented a phylogenetic tree and domain structure of 65 human Rho‐GAPs. However, ARHGAP42 showed a very low expression frequency in each cancer tissue (not including NPC). A previous study has identified ARHGAP42 as a Rho‐specific GAP expressed specifically in smooth muscle cells (SMCs) in mice and humans. The study has indicated that ARHGAP42‐mediated inhibition of RhoA activity in vascular SMCs is necessary to maintain normal blood pressure homeostasis.^12^

Of late, a lot papers have shown that the expression of many long noncoding RNAs is dysregulated in various cancers. These lncRNAs play important roles in tumor progression.^18^ LncRNAs have been confirmed to play a key role in gene epigenetic regulation, genome imprinting, X chromosome inactivation, and whole‐genome rearrangement.^19^, ^20^, ^21^ To the best of our knowledge, few differentially expressed lncRNAs have been reported to be associated with the tumorigenesis and progression of NPC. One example is H19, which is expressed at high levels in a human NPC cell line. H19 plays an important role in NPC cell differentiation and transcriptional silencing of imprinted genes.^22^, ^23^ The expression of antisense lncRNA has been shown to be correlated with repression in cis of protein‐coding genes. These genes spread over several hundred kilobases on either side of the antisense transcription.They indicated a link between lncRNA expression and silencing of neighboring protein‐coding genes.^24^

Antisense lncRNAs may form sense‐antisense pairs by pairing with a protein‐coding gene on the opposite strand and subsequently regulating epigenetic silencing, transcription, and mRNA stability.^25^, ^26^, ^27^

The present study provides the first description of ARHGAP42 as an NPC progression marker. Furthermore, we found that uc010rul decreased the protein level of ARHGAP42 but not the mRNA level of ARHGAP42, thus indicating that uc010rul may inhibit ARHGAP42 at a post‐transcriptional level. Natural antisense lncRNAs regulate sense gene expression through different mechanisms, such as transcription collision, DNA methylation modification, RNA splicing, and mRNA translation.^28^, ^29^, ^30^ The most common pattern of natural antisense lncRNA regulation on sense genes may be inhibition of the sense gene translation process through specific secondary structure. Other reports have shown that natural antisense lncRNA also affects chromosome stabilization through DNA methylation modification and histone acetylation. One study has revealed the existence of an antisense lncRNA capable of activating Zeb2 expression, explaining the mechanism involved in this activation and demonstrating that this antisense lncRNA regulates E‐cadherin expression.^31^

According to our findings, the role of uc010rul in regulation of ARHGAP42 may occur via the inhibition of mRNA stabilization or interference with the ARHGAP42 gene translation process. Further researches are needed to determine the correlation between the lncRNA uc010rul RNA level and ARHGAP42 protein expression in different patients with NPC. The ARHGAP42 gene cluster, including the natural antisense lncRNA uc010rul, might serve as a cancer progression marker in patients with NPC. We may further explore the mechanism underlying the regulation of ARHGAP42 by uc010rul.

In summary, the present findings provide evidence for a role of the ARHGAP42 gene cluster, including a role of the natural antisense lncRNA uc010rul, in metastasis of NPC cells. ARHGAP42 is a tumor migration marker and may be a prognostic factor or therapeutic target for patients with nasopharyngeal carcinoma. Together with previous work, our study highlights ARHGAP42 and uc010rul as novel targets for NPC therapy that may facilitate future nasopharyngeal carcinoma diagnosis and targeted therapy research.

## CONFLICT OF INTEREST

None declared.
