# Correction to “ARHGAP42 Promotes Cell Migration and Invasion Involving PI3K/Akt Signaling Pathway in Nasopharyngeal Carcinoma”

**DOI:** 10.1002/cam4.70559

**Published:** 2025-01-10

Q. Hu, X. Lin, L. Ding, et al., “ARHGAP42 Promotes Cell Migration and Invasion Involving PI3K/Akt Signaling Pathway in Nasopharyngeal Carcinoma,” *Cancer Medicine* 7, no. 8 (2018): 3862‐3874, https://doi.org/10.1002/cam4.1552.

Concerns were raised by a third party regarding overlapping image panels within the article (Figures 4D and 6E) and image duplication between this article (Figure 3D) and a previously published article by a different group of authors [1].

**FIGURE 3:**
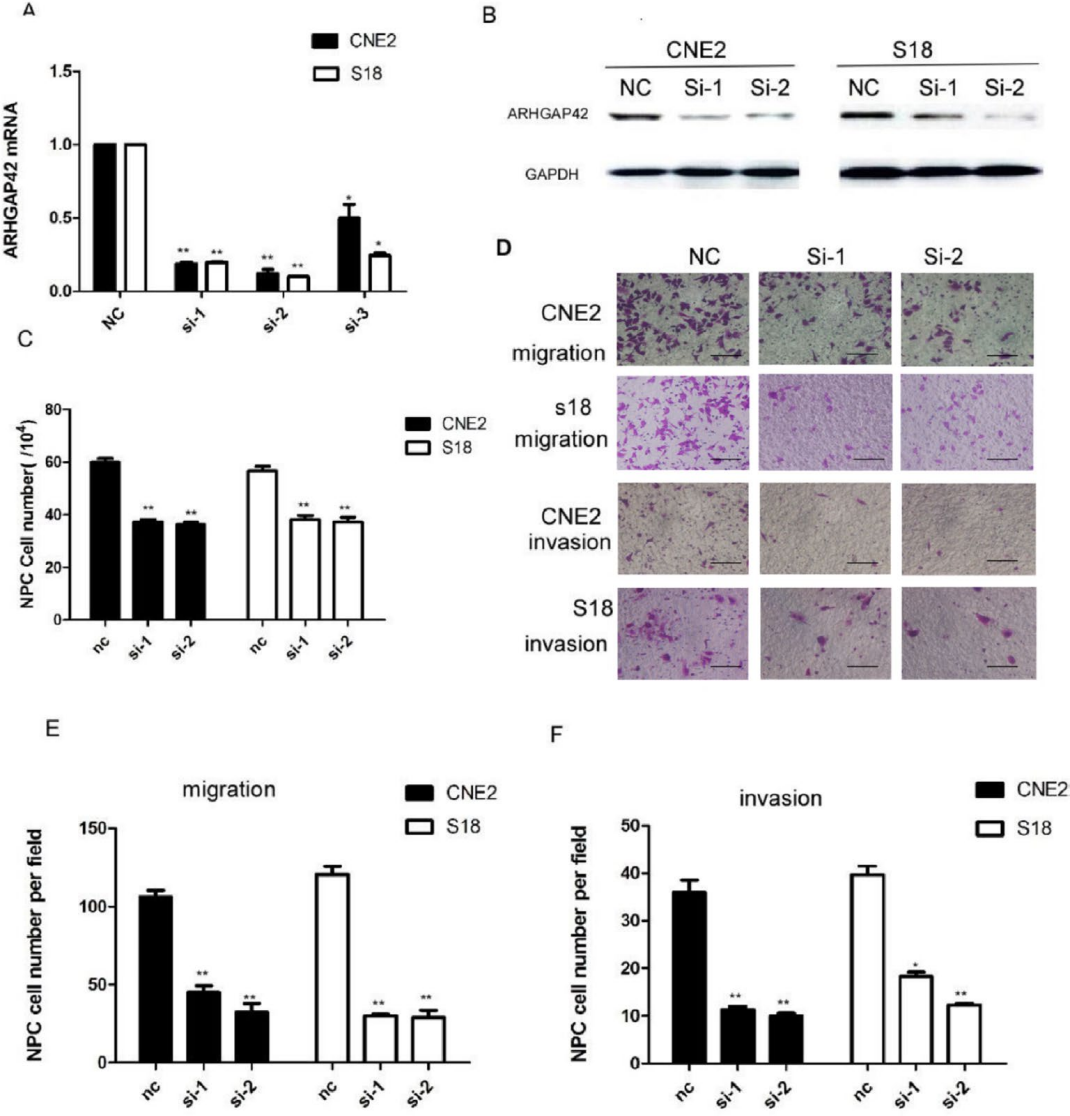
Knocking down of ARHGAP42 by siRNA resulted in significant inhibition of cell proliferation and mobility of S18 and CNE2. (A) ARHGAP42 mRNA expression in the NPC cell lines CNE2 and S18 by siRNA‐ARHGAP42 was measured by quantitative real‐time PCR, normalized to GAPDH gene expression. *p < 0.05, **p < 0.01. (B) ARHGAP42 protein expression in the NPC cell lines CNE2 and S18 by siRNA‐ARHGAP42 was measured by western blotting. (C) Knocking down of ARHGAP42 by siRNA resulted in significant inhibition of S18 and CNE2 cell proliferation by cell counting test in 72 h. *p < 0.05, **p < 0.01, compared with the negative control group. (D) Knocking down of ARHGAP42 by siRNA resulted in significant moveability inhibition of S18 and CNE2 cell lines, scale bar: 50 μm. (E) Knocking down of ARHGAP42 by siRNA resulted in significant inhibition of S18 and CNE2 cell migration. **p < 0.01, compared with the negative control group. (F) Knockdown of ARHGAP42 by siRNA resulted in significant inhibition of S18 and CNE2 cell invasion.

The authors admitted to the image compilation error in Figures 4D and 6E and the unintentional reuse of a previously published image for the s18 migration—Si‐2 panel of Figure 3D. The authors stated that during the experimental work, the two author groups shared a laboratory and used the same computer for image storage which could have led to the error in retrieving and compiling the images. The authors cooperated with the investigation and were able to provide the comprehensive raw data of the article.

The research integrity office of the authors' institution has investigated the concerns, including the repetition of the relevant experiments by two independent research groups and recommended issuing a correction. The authors confirm that all the experimental results and corresponding conclusions mentioned in the paper remain unaffected and sincerely apologize for the errors and any confusion caused.

The corrected Figures 3 and 4 and their corrected captions are as follows:

**FIGURE 4:**
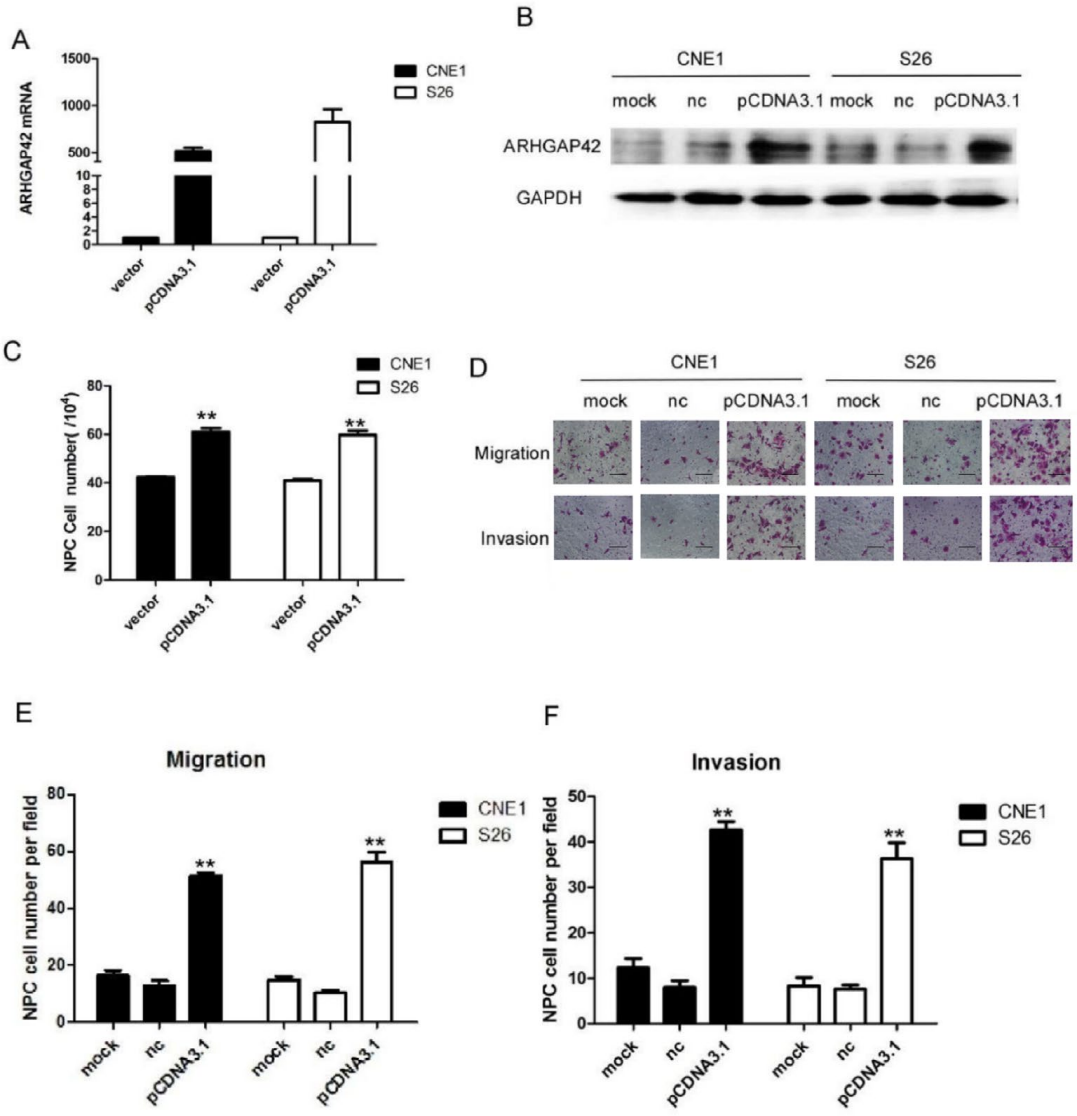
ARHGAP42 promotes cell proliferation and mobility. (A) Overexpression of ARHGAP42 mRNA was achieved in S26 cells and CNE1 cells by transfection of the ARHGAP42 expression vector. (B) ARHGAP42 protein expression level of CNE1 and S26 cells increased after transfection with the ARHGAP42 expression vector, as detected by western blot. (C) The cell counting test showed CNE1 and S26 cells were significantly increased after ARHGAP42 overexpression. (D) The migration and invasion of S26 cells were significantly increased after ARHGAP42 overexpression, scale bar: 50 μm. (E) Overexpression ARHGAP42 resulted in significant promotion of S26 and CNE1 cell migration. (F) Overexpression ARHGAP42 resulted in significant promotion of S26 and CNE1 cell invasion.

The authors also wished to make an additional change to Figure S1 where two patient samples showed overlapping sections. The correct images of the immunohistochemistry experiments displaying the immunohistochemistry staining for all patient samples can be found in Figure S1 of the revised Supporting Information.
