# Supplementary material for: ARHGAP42 promotes cell migration and invasion involving PI3K/Akt signaling pathway in nasopharyngeal carcinoma
Source: Cancer Med. 2018 Jun 24;7(8):3862–74. doi: 10.1002/cam4.1552 (PMC6089169; doi:10.1002/cam4.1552)

**Supporting Information**

**Table S1** siRNA nucleotides

| siRNA sense antisense |
| --- |
| uc010rul-si1 GCUGAGAUACGAAACGAUATT UAUCGUUUCGUAUCUCAGCTT  uc010rul-si2 GCAUCAUUGGUGUUUCAUATT UAUGAAACACCAAUGAUGCTT  uc010rul-si3 GCAUUAUCCGUGACCUCAATT UUGAGGUCACGGAUAAUGCTT  ARHGAP42-si1 GCAGAAUACAAGGAAUAAUTT AUUAUUCCUUGUAUUCUGCTT  ARHGAP42-si2 GCUCACCGGAAAUGUUUAATT UUAAACAUUUCCGGUGAGCTT  ARHGAP42-si3 GAGGAGUGAACUCCAAAGUTT ACUUUGGAGUUCACUCCUCTT |

**Table S2** PCR primers

| **Molecule sense primer antisense primer** |
| --- |
| ARHGAP42 5’-TGCTAACCAGGACTACAGACCACC-3′ 5’-CATTCATTTTCCCACTGGATTTCA-3′  uc010rul 5′-AAACTGCTGAGATACGAAACGAT-3′ 5′-CAACTTATGAAACACCAATGATGC-3′ |

**Table S3** Four NPC primary tissuesand three metastatic tissues from the nasopharyngeal carcinoma patients subjected to microarray analysis

| Patient | gender | Pathology | TNM staging | EBV-DNA（/copies） |
| --- | --- | --- | --- | --- |
| A | male | Primary Undifferentiated carcinoma | T3N2M0 | 0 |
| B | male | Primary Undifferentiated carcinoma | T3N2M0 | 4.7×10^6^ |
| C | female | Primary Undifferentiated carcinoma | T3N2M0 | 0 |
| D | male | Primary Undifferentiated carcinoma | T3N3M0 | 1.0×10^6^ |
| E | male | Dorsum metastatic Undifferentiated carcinoma | IV | 1.1×106 |
| F | male | Shoulder metastatic Undifferentiated carcinoma | IV | NA |
| H | male | Sternail metastatic squamous carcinoma | IV | 0 |

**Figure S1** IHC for a part of NPC patients

ARHGAP42 IHC staining in NPC tissue. Representative immunohistochemical expression of ARHGAP42 immunostaining in NPC tissues. Brown pointing at a dermal nest of stained cells (×100)


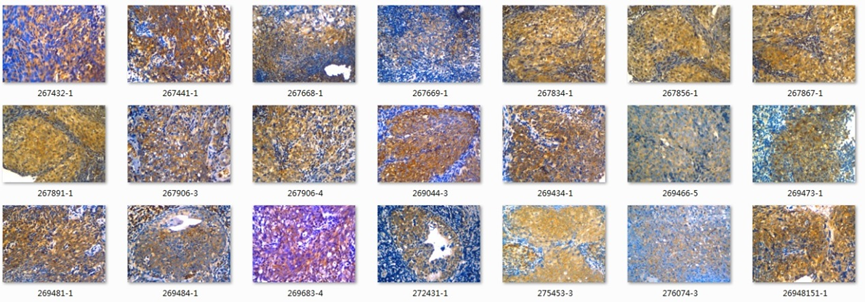

Supplement: Supplementary file 1 [file CAM4-7-3862-s001.docx]
